# Human-centred risk assessment for a land-based control interface for an autonomous vessel

**DOI:** 10.1007/s13437-022-00278-y

**Published:** 2022-06-15

**Authors:** Åsa S. Hoem, Erik Veitch, Kjetil Vasstein

**Affiliations:** 1 Department of Design, The Norwegian University of Science and Technology (NTNU), Trondheim, Norway; 2 Department of Engineering Cybernetics, The Norwegian University of Science and Technology (NTNU), Trondheim, Norway

**Keywords:** Risk assessment, Scenario Analysis, Human factors, Autonomous ships, MASS, Shore control centre, Shore control centre operator

## Abstract

Autonomous ferries are providing new opportunities for urban transport mobility. With this change comes a new risk picture, which is characterised to a large extent by the safe transition from autonomous mode to manual model in critical situations. The paper presents a case study of applying an adapted risk assessment method based on the Scenario Analysis in the Crisis Intervention and Operability study (CRIOP) framework. The paper focuses on the applicability of the Scenario Analysis to address the human-automation interaction. This is done by presenting a case study applying the method on a prototype of a Human–Machine Interface (HMI) in the land-based control centre for an autonomous ferry. Hence, the paper presents findings on two levels: a method study and a case study. A concept of operation (CONOPS) and a preliminary hazard analysis lay the foundation for the scenario development, the analysis, and the discussion in a case study workshop. The case study involved a Scenario Analysis of a handover situation where the autonomous system asked for assistance from the operator in a land-based control centre. The results include a list of identified safety issues such as missing procedures, an alarm philosophy and an emergency preparedness plan, and a need for explainable AI. Findings from the study show that the Scenario Analysis method can be a valuable tool to address the human element in risk assessment by focusing on the operators’ ability to handle critical situations.

## Introduction

Maritime Autonomous Surface Ships (MASS) are said to have a considerable impact on the shipping industry’s sustainability, promising greener and safer solutions (e.g. Fan et al. (2020); Porathe et al. (2018)). However, because it will change the way work is done, the chance is that it will introduce new risks. Technological developments within software and hardware have led to rapidly increased automation in many systems and applications. IMO (2019) defines MASS as a ship which, to a varying degree, can operate independently of human interaction. IMO distinguishes four degrees of autonomy: (1) crewed ship with automated processes and decision support; (2) remotely controlled ship with seafarers on board; (3) remotely controlled ship without seafarers on board; and (4) fully autonomous ship. The MASS concept entails not only the ships in themselves but the complex socio-technical systems consisting of equipment, machines, tools, technology, and work organisation. Human operators have different roles and interactions with ship systems and functions in each of the listed degrees.

The degree/level of autonomy will vary in a dynamic way between full human-operated control and full machine control. This dynamic autonomy brings an additional layer of complexity to the systems and operations, especially regarding the interactions and handover between human operators and autonomous technology. For the foreseeable future, a human operator must in some way be “in the loop”, supervising the operation and on stand-by to take over control from a land-based control interface referred to as a shore control centre (SCC). Still, most of the research on MASS focuses on technical components of the system, running a risk of missing the critical human element in MASS operations.

In a study on the influence of human factors on the safety of a remotely controlled vessel, Wróbel et al. (2021) identified the shore control centre operators’ (SCCO) condition and their ability to correct known problems, to potentially have the most significant influence on the occurrence of accidents. The study also indicates that the SCCO’s action represents the final and most important barrier against accident occurrence. Designing such a system should follow principles for meaningful human control (Hoem et al. 2021; van den Broek et al. 2020) and socio-technical design, like involving the future users of the new systems, in the interim guidelines for MASS trials (IMO, 2019), the International Maritime Organization (IMO) stipulate that “for the safe, secure and environmentally sound conduct of MASS trials, the human element should be appropriately addressed.” In IMO’s guidelines for Formal Safety Assessment (FSA), it is stated that “the human element is one of the most important contributory aspects to the causation and avoidance of accidents…. Appropriate techniques for incorporating human factors should be used” (IMO 2018a). FSA is commonly seen as the premier scientific and systematic risk assessment approach. Per the latest revised guidelines (IMO 2018a), the FSA consists of five steps: (1) identification of hazards; (2) risk analysis; (3) risk control options; (4) cost–benefit assessment; and (5) recommendations for decision-making.

Risk definition and perspectives in the maritime domain are strongly tied to probabilistic methods (Goerlandt and Montewka 2015). This classical approach to risk analysis involves a process governed by data collection, processing, and calculating quantitative risk metrics using engineering and inferential statistics. Risk analyses are well established in situations with considerable data and clearly defined boundaries for their use. However, for MASS, we do not currently have sufficient empirical data. In addition, the complex and software-intensive technology of MASS, composed of not only hardware components but also logic control devices and a high number of sensors (Zhou et al. 2020), makes an accurate quantitative risk estimation extremely difficult to achieve. Furthermore, if achieved, the uncertainty related to these numbers will be high. Literature on risk assessment of MASS acknowledges the lack of data on design solutions and system architectures (Hoem 2019), making it challenging to apply probabilistic risk assessments. There are, however, arguments for seeing beyond expected values and probabilities in defining and describing risk. Over the last 20 years, there has been a shift from narrow perspectives based on probabilities to assessing a broader risk picture reflecting different views, assumptions, and ways of thinking that highlight events, consequences, and uncertainties (Aven 2009; 2012).

Risk assessment should be carried out both during the design and operation of MASS (Utne et al. 2017). During operation, risk monitoring and control are carried out both by the SCCO and by the technical system. MASS will have online risk control functionalities implemented in its control system, as described by Utne et al. (2017). In addition, the system shall visualise the risk monitoring through the HMI to the SCCO to support decision-making when human intervention is needed (for example, by real-time indicators on the systems’ status, weather conditions, and presenting detected objects within the collision zone). With the integration of the SCC, interaction-associated hazards may lead to accidents if not well recognised and controlled (Yang et al. 2020).

Risk assessments in the design process are tools for decision-making. They can broadly be used in two ways: formative analyses (focused on the process, e.g., to improve the quality of a design) or summative (focusing on the results of the assessment, e.g., to evaluate if a safety target is met) (French et al. 2011; French & Niculae 2005). Some of the main reasons for carrying out a risk assessment are listed in Table 1 below. The activities represent some of the different phases of a product development cycle.

**Table 1:** Formative and summative use of risk assessments

Activities	Formative analysis	Summative analysis
Design	Proactively used to “design out” potential system failures and issues	Used to verify the capabilities and performance of the technology
Regulation and approval	Helps to choose between possible solutions	Demonstrates compliance and that a safety target is met
Licensing and verification	Helps understand modifications of the current design	Demonstrates fulfilment of a performance standard

### Risk-based ship design 

According to IMO and current best practices and regulations, MASS will be approved according to principals for alternatives and equivalents (IMO 2013). This is fundamentally a *risk-based* approach rather than a *rule-based* approach where operational or functional requirements must comply with the statutory rules and regulations. The regulatory framework for risk-based ship design (RBSD) was introduced with the primary objective to provide evidence on the safety level of a specific design of ships (Papanikolaou and Soares 2009), i.e. a summative approach to risk assessment. Meeting a particular level of safety (predefined risk acceptance criteria) implies that safety must be quantified using a formalised quantitative risk analysis procedure.

RBSD framework has mainly been applied for technical design (Ventikos et al. 2021). Applications including human element considerations are relatively fewer. This is most likely because guidelines on RBSD, such as Lloyds Register’s procedures on risk-based design (2016) do not provide any guidance on including human and organisational aspects of risk. However, as IMO (2018b; 2019) states, the human element should be assessed as a part of an FSA and risk analysis in the design of MASS.

In the RBSD methodology, the human element is considered a factor that influences the causation probability. Quantifying the human element’s contribution to risk is typically done by applying a human reliability analysis (HRA) method. HRA focuses on “human errors” as a cause. However, the “New view” of “human error” (Dekker 2014) focuses on “human error” as a symptom of problems rather than a source/cause of them. Typical problems are poor design or organisational issues. The classical view of “human error” is criticised by many as too narrow (e.g. Boring et al. (2010); Dekker (2014); Hollnagel (2000); Leveson (2016)). Quoting Leveson (2011), although the human element rests in the centre of socio-technical systems and guarantees their sustainability and viability, humans are seen as components with defined specifications. Hence, “human error” becomes a local problem rather than a symptom of flawed designs. Leveson (2011) presented a System Theoretic Process Analysis (STPA) method where “human error” is examined in its context, unsafe control actions, and control mechanisms that shape human behaviour. Many researchers see the STPA as a promising risk assessment to be applied to MASS concepts (Banda et al. 2019; Thieme et al. 2018; Utne et al. 2020; Wróbel et al. 2017, 2018; Zhou et al. 2020). However, this top-down method requires a hierarchical safety control structure, both on technical and organisational design (see Leveson and Stephanopoulos (2013)), making the analysis complex and involving many steps that are not easy to follow or understand (Hirata & Nadjm-Tehrani 2019). The control structure is dependent on what is included in the system and where the system boundary goes. For engineering, the most useful way to define the system boundary for analysis purposes is to include the parts of the system over which the system designers have some control.

### Risk-informed decision-making in the design of MASS

**Fig. 1:**
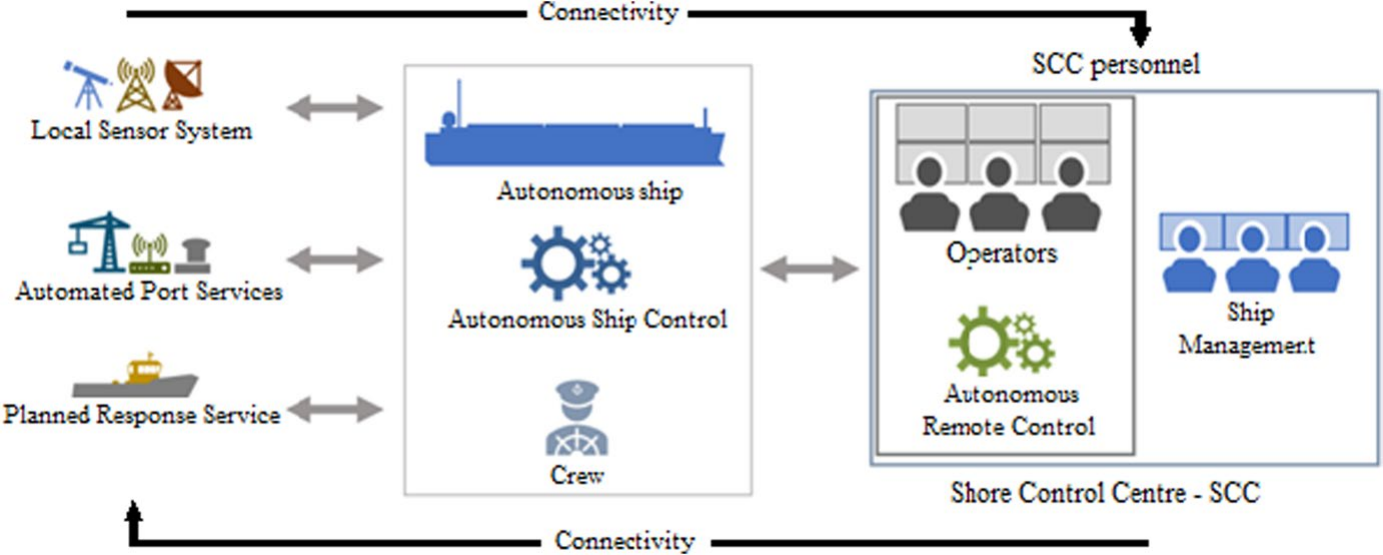
Examples of components and roles in an autonomous ship system, adapted and adjusted to the content of this paper from Wennersberg et al. (2020)

In RBSD, only the ship is considered. For MASS, as mentioned, the vessel will be part of a more extensive system involving different components and actors, as shown in Fig. 1 below.

In the context of this paper, “risk-based design” simply means carrying out risk analyses that are not necessarily quantitative in the design process. The term *risk-informed decision-making in design* may be a better phrase for explaining our approach. Papanikolaou and Soares (2009) have already described a similar risk-based design approach based on probabilistic functional requirements. Risk-based decision-making is criticised for focusing too much on probabilistic risk estimates and paying too little attention to design principles (Rausand 2013). Risk analysis is not the same as decision-making but is merely one tool in the process.

The Norwegian Maritime Authority NMA (2020) and classification societies, such as Bureau Veritas (2019), ClassNK (2020), DNV (2018), and Lloyd’s Register (2017), have published guidelines on risk assessments of MASS. They all recommend applying a risk-based approach. DNV (2018) states that the design methodology should specifically address all functions of the auto-remote infrastructure needed to achieve an equivalent level of safety. The guideline mentions, explicitly, the CRIOP study as a risk analysis method focusing on human aspects. Hoem et al. (2021) presented an adapted version of the framework as an interdisciplinary risk assessment method in designing a SCC for the operation of MASS.

The CRIOP framework is an established, standardised scenario method primarily developed for the oil and gas industry in 1990 (Johnsen et al. 2011). Since then, the methodology has developed through collaborations between regulatory authorities, operators, research institutions, contractors, and consultants to include and consider HMI, best practices standards, and human factors, including principles from the ISO^9241^-^210^ (2019) and ISO11064 (2013) standards and the barrier management perspective (Johnsen et al. 2020). Today, CRIOP is used to verify and validate a control centre’s ability to handle all operational modes safely and efficiently, i.e. normal operations, maintenance, disturbance/deviations, and safety–critical situations. The key elements of CRIOP are checklists covering relevant areas in the design of a control centre, scenario analysis of critical scenarios, and a learning arena where the operators, designers, and managers can meet and evaluate the optimal CC (Johnsen et al. 2011). The CRIOP process consists of four major work tasks:

1. **Prepare and organise** by defining, gathering the necessary documentation, establishing an analysis group, identifying relevant questions and scenarios, and setting a schedule.
2. **General analysis (GA)** with checklists to verify that the CC satisfies the stated requirements based on best industry practice (a standard design review).
3. **Scenario analysis** of critical scenarios. A cross-disciplinary team, including the end-users, perform the analysis to validate that the CC satisfies the actual needs.
4. **Implementation and follow-up:** At the end of tasks 2 and 3, the findings and recommendations are documented, and an action plan is established.

The scenario analysis’s third work task is designed to verify that the control room operator (CRO) can perform the required task while considering cognitive abilities, human-system interaction, and other performance shaping factors. The analysis is human-centred, focusing on the CRO’s interaction with the system, including communication with other personnel. Emphasis is on how the systems support the operator’s situation awareness and decision-making in different situations.

The analysis considers a few relevant scenarios, identified as key scenarios. The methodology suggests using Sequentially Timed Events Plotting (STEP) diagram for a graphic presentation of each scenario and its events. Considering each event involving the operator, questions like “what can go wrong?” and “what if?” are asked to identify potential hazards and safety issues. Additional questions related to the simple model of cognition (by Hollnagel (1996)) can be asked to determine how the systems support the operator’s situation awareness and his/her ability to make decisions and execute actions.

Furthermore, checklists on performance shaping factors are also used for additional questions to elaborate on the answers received. The questions and checklists help identify so-called weak points. Weak points comprise an identification of possible conditions, design issues, or safety problems in the achievement of operator tasks (involving identification, interpretation, planning and action on a situation). After identifying weak points, an evaluation of possible barriers and mitigating measures is initiated, and the results documented.

A CRIOP study can be applied at different phases of the design process. In the preliminary design, when the detail level is low, the method can assist in evaluating the assigned responsibilities between the autonomous system and the human operator. At this early design stage, it can also assist in identifying risks and ensuring end-user/operator involvement. At the final design stage, a CRIOP study can function as a tool for verification and validation by assuring the quality of documented task analyses, workload analyses, work environment/ergonomics, quality of alarms, and HMI (Johnsen et al. 2020).

### Problem description and main ideas

The current RBSD framework is not adjusted for the design of MASS (including its integration with a SCC). For the risk-based design of MASS, the summative classical approach will be challenging for practical use as the background knowledge—the basis for the probability models and assignments—is weak, i.e. uncertain. The term uncertainty is used to capture the idea that a person or group does not know the true value of a quantity or the future consequences of an activity due to imperfect or incomplete knowledge (Aven 2019). In the face of uncertainties, the risk assessment of MASS may be better addressed by constructing scenarios that are validated according to logical consistency, psychological empathy with the main players involved, congruence with past trends, and narrative plausibility (see Aven and Renn (2009)). The main players involved in the operation of MASS are, as mentioned, the critical human element, i.e. the SCCO. However, few risk assessment methods address the SCCO in the design of MASS today (Veitch and Alsos 2022), and the classical technical risk assessment methods are insufficient to address human-automation interactions (Goerlandt 2020). Two recently developed methods, the STPA (Leveson, 2016) and *Human System Interaction in Autonomy* (Ramos et al. 2020), require a high level of system knowledge and method expertise. In addition, they can be quite time- and resource-consuming, making them of limited value in an early design phase when developing an HMI for a SCC.

Veitch and Alsos (2021) present a human-centred SCC design approach and bring in the concept of resilience by addressing the safety–critical interactions between the SCCO and the HMI. The authors acknowledge the need for building on this idea and use a systematic risk assessment method like the scenario analysis used in the CRIOP framework. This paper presents a case study where the adapted scenario analysis is carried out on an actual first prototype of an HMI for a SCC. The overall research question is as follows: can the scenario analysis support risk-informed decision-making in the design of a SCC?

This paper presents a method for carrying out a human-centred risk assessment and a use case where the method is applied in a workshop. The following section presents the scenario analysis methodology and the findings in a literature study of the method. Section 3 describes how we carried out a case study (the format of the case study-workshop, the HMI simulator, and the preparations) and the results, followed by a discussion of the applicability of the scenario analysis as a risk assessment tool in Section 1.3 and a conclusion in Section 2.

## A review of the CRIOP scenario analysis 

Hoem et al. (2021) presented how the scenario analysis in the CRIOP Framework (Johnsen et al. 2011) can be adopted, aiming to identify hazards and risks, assess them by identifying weak points in the design, evaluate existing barriers, and develop measures (mitigation actions) to improve the design. This section reviews the CRIOP scenario analysis in light of its contributions to risk analysis and design research.

We examined the research question by further asking in-depth questions like how the scenario analysis supports the idea of having the “human in the loop” during both design and operation and how the scenario analysis contributes to the FSA framework. Furthermore, what are the benefits and limitations compared to other risk analysis tools?

**Fig. 2:**
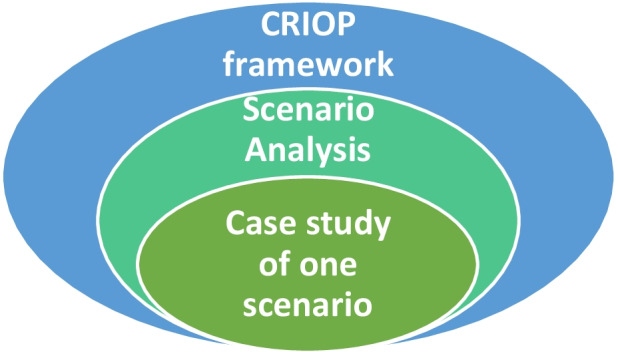
The scope of the paper is a case study of a scenario analysis adapted from the CRIOP methodology

A case study (further discussed in Sect. 3) applying the scenario analysis method was carried out in the setting of a CRIOP-workshop, as shown in Fig. 2. We wanted to evaluate the applicability of the adapted scenario analysis and explore the contextual conditions of the method.

The adapted scenario analysis method can be summarised in the following steps in Table 2.

**Table 2:**
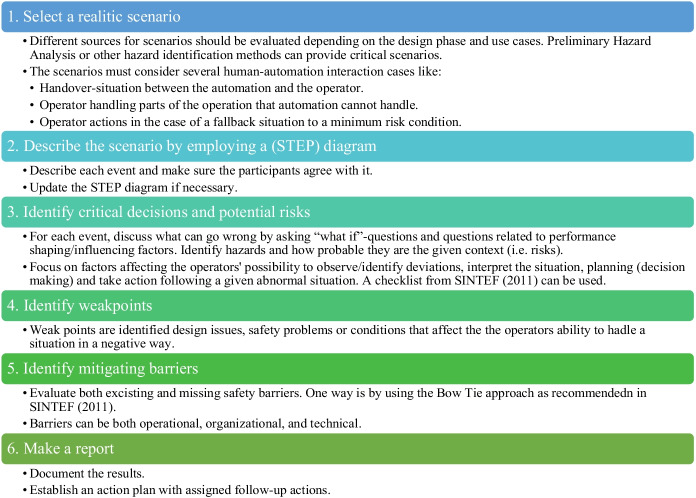
The main activities of the adapted scenario analysis inspired by the CRIOP Framework

Steps 1 and 2 should be carried out prior to a workshop by experts from different fields of expertise. The scenario analysis group should consist of designers, end-users, engineers, software developers, human factors experts, and management. Depending on the scenario, it could also involve a broader range of stakeholders. By involving people with experience from similar systems and including the end-users, the analysis aims to minimise the gap between work as imagined (WAI) and work as done (WAD), considering the resources needed to execute the operations. WAI refers to the various assumptions, explicit or implicit, that people have about how work should be done. WAD refers to (descriptions of) how work is actually done, either in a specific case or routinely (Hollnagel 2017).

The purely technology-centred approach can sometimes lead to structural and functional rigidity in the design and operation processes. The consequences are that people must adapt to the system, and the HMI is something that is put on top of the system in the end after it has been built, which is the opposite of human system integration or human-centred design (HCD). This is also an issue of WAI vs WAD. In a scenario analysis, we are at the blunt end considering work as imagined (WAI). We use our expectations based on our experience of actual similar work (at the so-called sharp-end). It is practically impossible to predict or describe how work is done by others since it occurs at a different time and place (Hollnagel 2017). However, we can imagine how work is to be done and why in abstract terms. By including the actual end-user and presenting the scenario for them at their workstation, we can discuss if the imagined work is as close to the “work as done” as possible and be aware of the actual difference. As mentioned by Lützhöft (2004), people participate in integrating new technology into complex fields of practice—often in ways that are surprising to the designer, and involving the prospective users is necessary for providing knowledge of the current practice.

Furthermore, we avoid defining the design needs based solely on abstraction by carrying out scenario analyses at different stages of the design process (i.e. after the conceptual/preliminary designed HMI, the detailed design, and the built HMI). In this way, the method presents an opportunity to improve our models of work throughout the design.

The scenario analysis should be used as a formative method that recognises and roughly rank the potential for improving safety issues (i.e. the weak points) related to the HMI. The method can help improve the design of the HMI itself, the structure of the organisation, and the processes by which it is operated. Researchers have pointed to the need for bringing in human factors expertise early in the design process, e.g. Blackett (2021) and Johnsen and Porathe (2021), to avoid poorly designed solutions that are challenging and costly to change. The design process should be iterative and involve human factors and the end-user from the beginning, to support sensemaking and meaningful human control.

### An iterative human-centred risk assessment

**Fig. 3:**
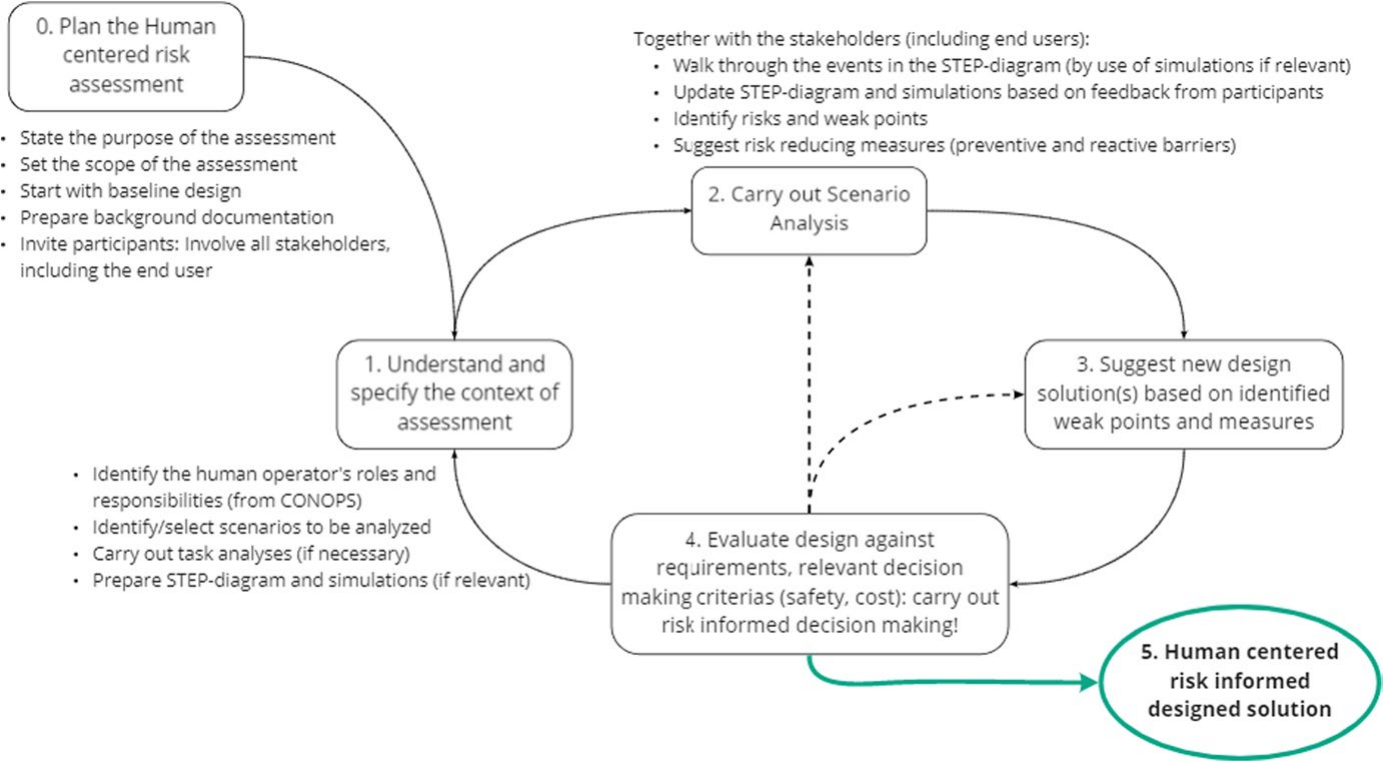
The iterative human-centred risk assessment approach based on the HCD process (ISO9241-210 2019)

The CRIOP exercise, and hence the scenario analysis, is a participatory (multidisciplinary) iterative process. The methods support the HCD process activities for interactive systems (ISO^9241^-^210^,^2019^). By applying an HCD process, flexible and robust design solutions might be achieved where the operator situation awareness recovery, task switching support, and workload balancing are considered. The scenario analysis may work as both an analytic (in analysing the human–machine interactions) and an evaluative tool (evaluating the design against requirements). However, in the case of designing an HMI for a SCC, we do not have any predefined acceptance criteria available to measure a prototype against yet. The adapted scenario analysis can be considered a dual view approach to risk analysis, as both objective facts and subjective statements are considered. The result of a scenario analysis is a list of weak points and suggestions for improvements (i.e. mitigating barriers) and not a complete characterisation of a risk or a risk picture. The overall goal of a scenario analysis is to improve the design by enabling human-centred risk-informed decision-making. The adapted scenario analysis as a human-centred risk assessment process is visualised in Fig. 3 below.

### Identifying hazards and safety issues not covered by existing risk analyses

Looking at what can fail or go wrong is a bottom-up approach whereby safety is treated as a failure prevention problem. Applying this methodology to complex systems has, in recent years, spurred vocal criticism (see, for example, Leveson (2020)). A hazard analysis is traditionally seen as a failure and malfunction of components. This is not necessarily the same as asking “what can go wrong?” and “what if?” questions. These questions imply that something surprisingly can happen due to a combination of performance variabilities.

The scenario analysis allows the participants to take the SCCO’s role and experience how incidents and accidents can be handled based on available information presented to the SCCO. Hence, unlike many risk analysis methods, the scenario analysis focuses on the operational experiences of the HMI. Hazards and potential safety issues that are not necessarily revealed by traditional risk analysis can be identified by focusing on the SCCO’s responsibilities, tasks, and capabilities. In this way, issues related to a poorly designed solution, a lack of explainable AI (see Veitch and Alsos (2021)), or deficient procedures and responsibility can be identified as weak points and handled early.

Like most traditional hazard analysis techniques, the STEP diagram provides a chain of events, addressing factors that affect how the accidental events are presented to the operator and propagate. The aim is not to track accidents back to a root cause or identify component failures but rather how different actors (including the end-user) experience a scenario sequence and which control and interaction issues may arise. In a setting where humans can brainstorm on possible interaction issues, the direct linear causality of the deterministic cause-effect relationship does not affect the risk analysis like a typical on paper risk model would. We can think more abstract than we can write down in a 2D model. The identified hazards, mitigating measures, and weak points do not need to be directly connected to an event in the STEP diagram.

The STEP diagram demonstrates how operational scenario sequences might be unambiguously specified by getting the workshop participants’ second opinion. Furthermore, presenting the course of the scenario in a STEP diagram brings up an agreement between the designers, engineers, end-users, and the software developers on how the “behaviour” of the technical system and the SCCO’s action should enfold, and hence (if necessary) redefine the system architecture at an early stage. The STEP diagram can further be translated into an event sequence diagram and give input to task analyses used in more advanced and comprehensive safety analyses, like the *Human System Interaction in Autonomy-*method proposed by Ramos et al. (2020).

Hazards and risks are in the scenario analysis considered in “two turns” by selecting scenarios based on a preliminary hazard analysis and further in the STEP diagram by asking what can go wrong, focusing on the SCCO’s capabilities. This allows us to dive deeper into the challenging parts of the HMI and question how the HMI can support the SCCO to have quick detection and early response to a critical situation.

The method supports the underlying idea of resilience as the ability to sustain or restore its basic functionality following a stressor (Hollnagel et al. 2006). Increasing the resilience can be seen as a strategy for managing risk. In this case, we design for a safe and resilient HMI by identifying hazards and weak points and subsequently risk-reducing measures focusing on the SCCO’s capabilities at an early stage. As recommended by Aven (2016), by applying a scenario-based risk analysis focusing on the capabilities of a SCCO, we are relating risk to performance and hence incorporate resilience dimensions.

### Compared to the system theoretic process analysis 

There are many risk assessment methods available to the designer. Their applicability depends on the purpose of the risk assessment (whether, for example, it is used to decide if an activity should be permitted, if a system is safe enough, if system improvements are necessary, or simply in choosing between competing options). For MASS, the effectiveness of the risk assessment varies with respect to different autonomous system properties (Bolbot et al. 2020). Zhou et al. (2020) have investigated the applicability of 29 hazard analysis methods for autonomous ship systems. The scenario analysis was not evaluated, as it is not targeted at ships or applied within the maritime domain. However, the adapted scenario analysis fulfils many of the evaluation criteria (EC) listed in the review (see Table 3 in Zhou et al. (2020)). The method can be used to analyse system-level hazards (EC1); can be used from the early system development phase (EC2), can be used to analyse the hazards resulting from HMI (EC6); consider the communication between ships and SCC (EC7); consider the communication among shore-based operators, or crew onboard (EC8); and consider different operational modes resulting from the change of levels of autonomy (EC10). The STPA fulfils all criteria listed in Zhou et al. (2020), and because several authors have recommended it as a promising method for risk assessments of MASS, it was selected for further comparison.

**Table 3:** The characteristics of the participants in the online workshop

Participant no	**Disciplines and experiences**							
	Safety engineer	Naval architect	Interaction design	Marine cybernetics	Engineering cybernetics	Mariner w/ seagoing experience	Control room experience	Member of the SCL
1 M	○		○			●		x
2 M			●			●	○	x
3 M	●		○				●	
4 M			●					x
5 F			○				●	x
6 M				●		○		
7 M		●		●				
8 M				●				
9 M		●						x
10 M					●			
11 F					●		●	
12 F	●		○				○	x

Banda et al. (2019) applied the method to carry out a hazard analysis in the concept design of autonomous passenger ferries. The study presented a systematic hazard analysis based on the STPA framework. However, a SCC was not part of the analysis. Still, several identified safety control actions were suggested involving a SCC to communicate with the passengers or remote monitoring and fault detection of the technical systems. All the identified hazards are related to the technical system (i.e. component failures), the environment (heavy weather, strong current), or the passengers on board (falling/jumping overboard, medical conditions, etc.). In the selected cases (two urban passenger ferries), it was not addressed who is responsible for the safety of the passengers when the vessels are in operation. The reason why the SCC was left out of the scope seems to be that the suggested design process adopts the foundations of the ship design spiral, a 60-year-old design concept without any human factor considerations, hence, neglecting an essential source of risks and operational issues.

STPA does not define any framework for an operational scenario-based analysis, although STEP diagrams could be used in such an analysis. In the STEP diagram of a critical scenario, the events that involve crucial decisions by the SCCO can be seen as safety control actions applied in the STPA. Unsafe control actions (another term used in the STPA) are defined by asking what can go wrong here. In STPA, an unsafe control action is an action leading to an identified hazard, and typically when:

- A control action for safety is not provided or followed.
- A safety control is provided too early or too late
- A safety control is stopped too soon or applied too long
- A safety control is degraded over time
- An unsafe control action is provided

These aspects could beneficially be integrated into the scenario analysis and help define the scope and target of the analysis. As Zhou et al. (2020) suggest, possible combinations of the STPA and other risk assessment methods should be considered in future research. Explaining why and how these unsafe actions can occur is essential when identifying risks and weak points in the designed prototype. The result of an STPA is the list of accidents and hazards, the safety control structure, unsafe control actions, and causal factors. A scenario analysis can help identify the context that makes these results and the STPA claim that “the system is free from unacceptable risks leading to an accident” justifiable.

### The method’s contributions to the FSA framework

The scenario analysis method is in line with the FSA methodology. It can be seen as one framework to support the requirements of incorporating the “human element” in risk assessment, associating them directly with the occurrence of possible accidents, underlying causes, or influences (ref. Section 3.4 in IMO (2018a, b)). However, the scenario analysis does not introduce a risk matrix, or similar, to discuss the probability and consequences. The aim is to identify what could go wrong (identify hazards, events, and conditions that may lead to an accident or incident), how these may lead to different consequences, and suggest measures to avoid/limit the impact by focusing on the capabilities of the SCCO, hence, contributing to the majority of the steps in the FSA presented in Section 1.1.

The adapted scenario analysis is in line with the request for risk-based design (ref. guidelines listed in Sect. 1.1), where several different risk-analysing methodologies are utilised. The method can be considered a risk analysis associated with the remote supervision and control of a MASS from a SCC, explicitly focusing on the SCC and its supporting systems (ref. DNV GL, 2018)). If the scenario analysis’s tabletop exercise is carried out in a systematic manner, the assessment can provide valuable documentation and verification of a risk-based design process. It can also be seen as a tool used to represent and describe the knowledge and lack of knowledge of the autonomous system, its performance, and interactions with the SCCO.

It is important to remember that the primary goal of a CRIOP study is not the identified hazards but the identified weak points and the measures to improve them (with a correlating action plan). The identified hazards and risks can provide a basis for arguments on the need for design modification and contribute to risk-informed decision-making. Hence, the method provides decision support and could help the design team choose between alternatives, adjust the SCCO’s activities, and implement risk-reducing measures, for example, in the case of a clear alarm philosophy or specific improvements for the HMI. In the design process of the SCC, the early scenario description and analysis exercise can also provide valuable discussions on how to balance operational complexity with technical simplifications.

## Case study of the CRIOP scenario analysis method

A SCC for the operation of an autonomous urban passenger ferry, the milliAmpere2,^1^ was the subject of the analysis carried out in a workshop with participants. The case study aimed to test the applicability of the scenario analysis framework by evaluating the validity, credibility, and reliability of the approach based on the exploration of a critical scenario in a simulated SCC together with experts from different disciplines. The goal of the scenario analysis was to improve the prototype HMI design by carrying out the risk assessment and identifying weak points (with suggested mitigating measures to improve them).

We used a qualitative case study methodology to do a process and outcome evaluation (Yin 2009). We have an initial descriptive theory about the case tentative to the study and a hypothesis about the expected characteristics of the case. We take an interpretive perspective to the case study by presenting our view as researchers on the scenario to be analysed (thus interpreting elements of the study). However, the approach is also relativistic as we aim to include the participant’s multiple perspectives on the method: How do they interpret the risk analysis method? Do they find the scenario analysis helpful in identifying weak points? Moreover, do they believe the method is a good tool for risk-based design?

**Fig. 4:**
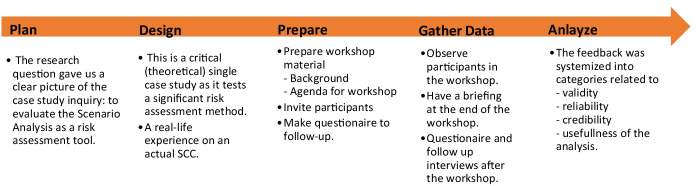
Our case study research approach with activities and sub-activities

The case study research process is shown in Fig. 4. Our descriptive framework for organising the case study should not be confused with the scenario analysis method. Notwithstanding this important distinction, there are some natural overlaps in activities such as inviting participants and collecting feedback.

### The SCC for remote operation of milliAmpere2

**Fig. 5:**
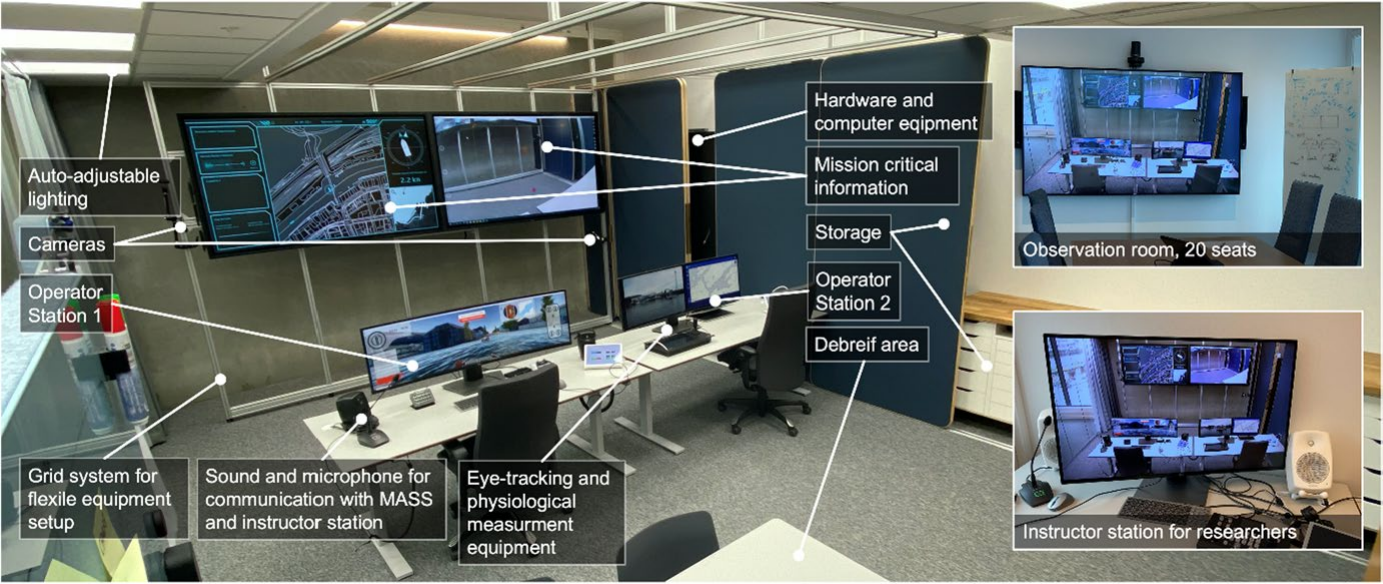
The Shore Control Lab (SCL) at the Norwegian University of Science and Technology (NTNU)

The shore control lab (SCL) (Fig. 5) is a test platform for research in highly automated ships. The lab is equipped with testing equipment to support research and development of the human control side of autonomous ships. One of the central research tools is a custom-built simulator based on the Gemini open-source platform (Vasstein et al. 2020). The simulator, built in Unity, allows for flexible testing in immersive environments with high-fidelity graphics and realistic physics engines.

**Fig. 6:**
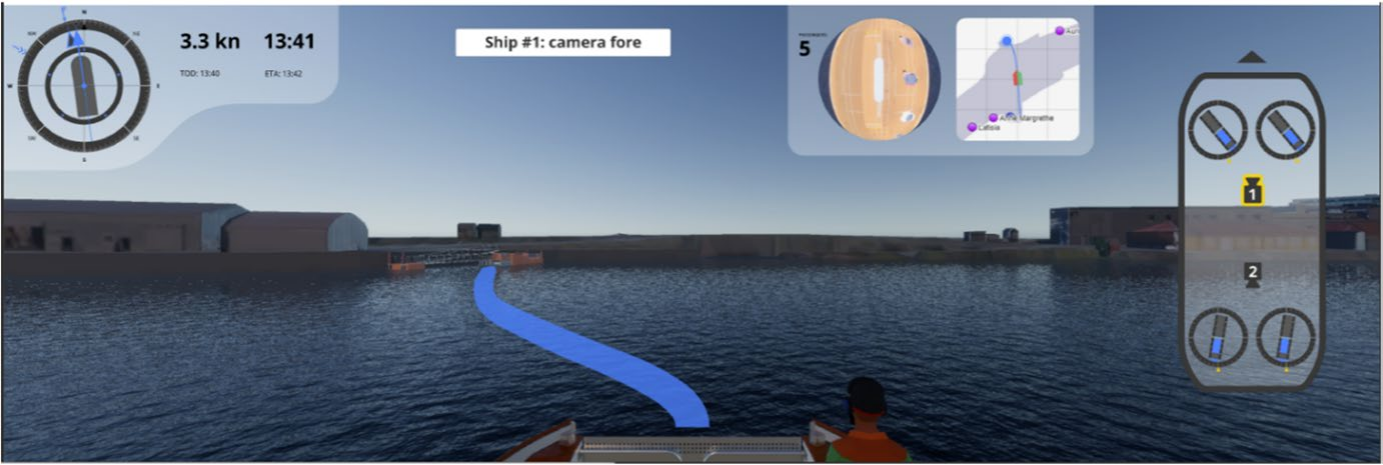
A screenshot of the simulator

The simulator presented in the workshop is illustrated in Fig. 6. The graphical user interface (GUI) displayed a simulated camera view from onboard the milliAmpere2 ferry in the approximate location where the physical camera is mounted. The GUI overlays show essential information like speed, heading, and the number of passengers. The central HMI consisted of a GUI and a control pad for handling actions (stop ferry and keep ferry in position by dynamic positioning (DP), drop anchor, manual control switch, communication with passengers, harbour authorities and emergency response, etc.). In addition, other peripherals like a joystick for manual control, keyboard, mouse, and speakers for alarms were available.

### Preparations before the workshop

A concept of operation (CONOPS) for milliAmpere2 was developed by zeabuz (2021). The SCC was not included in the scope of the CONOPS, as a safety operator (responsible for safeguarding the passenger and the operation of the ferry) would initially be present onboard the ferry. The safety operator onboard would be able to initiate a safe state (set the ferry on DP or drop anchor), use a VHF radio, contact the harbour and emergency services, and manually control the ferry. An incremental approach to moving the safety operator to a SCC is suggested in the CONOPS. Three elements informed the tasks and responsibilities of the SCCO: the design of the ferry (including the autonomous and automation system), the tasks envisioned for the safety operator, and the experience from other domains (i.e. a remote-control centre for offshore oil and gas installations). The design team at the SCL made a background document presenting the operational domain, the design of the ferry, the HMI at the SCC (with peripherals), the SCCO’s tasks and responsibilities, and the emergency organisation and response procedures. The document was an internal document shared with all participants before the workshop to build a shared understanding of the scope.

#### Selected scenario

**Fig. 7:**
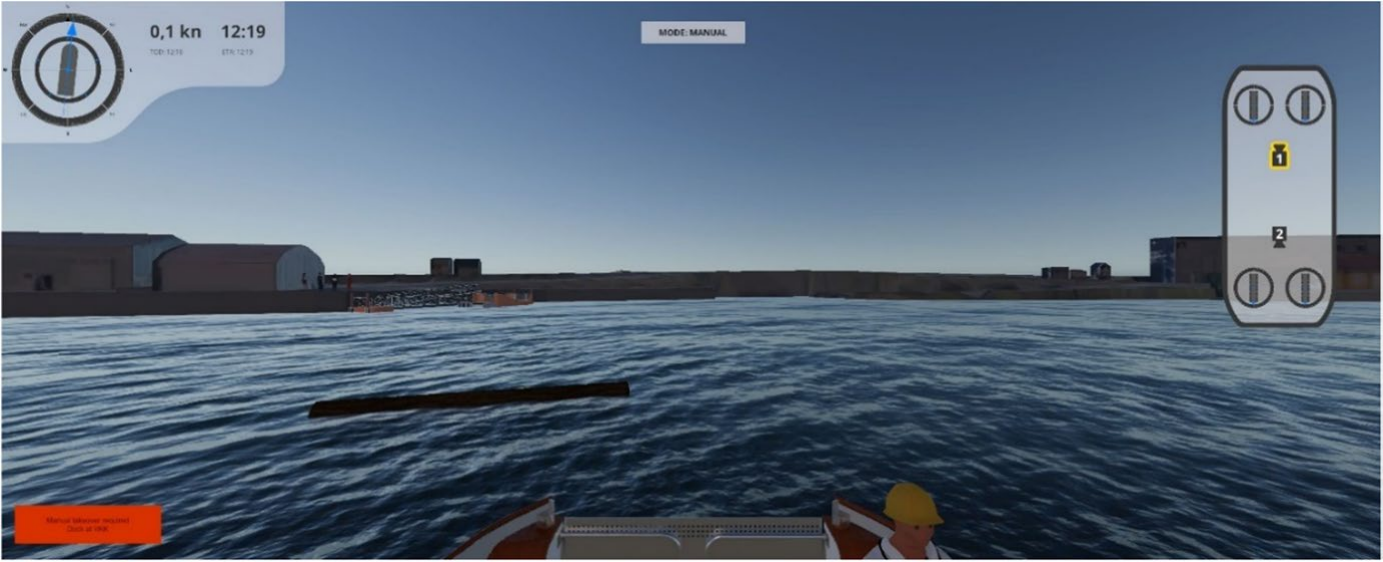
A screenshot of the GUI showing a partly submerged log in the pathway of the ferry

The design team arrived at a scenario where an unexpected object (in this case, a partly submerged log) was floating in the pathway of the ferry, causing the ferry to stop, stay in position (automatically activate DP), and send a notification to the SCCO to assess the situation (see Fig. 7 below). The SCCO can then take manual control by switching a button on the control pad. A notification message “Manual control engaged” will be visible in the lower corner of the GUI until the switch is turned back to “Resume autonomy”.

Before the workshop, a preliminary hazard analysis was carried out by the technology company zeabuz and facilitated by the classification society DNV. A list of critical situations was identified, one of them being the handover when the automated systems “ask” the safety operator to take over control. In a study by Dybvik et al. (2020), designing the HMI was identified as the most challenging part of a SCC design. In particular, this involves the handover from automation to human control. Knowing how to resolve this situation is a design issue and key to designing the interface. The simulator tool presented in the previous section made it possible to explain such a handover situation. The version shown here was specifically designed to confront users with a handover situation involving a shift of control from the automated system to the remote operator that occurred unbeknownst to the user-tester. The best GUI design, one that supplies the relevant information to the operator in the most appropriate way, will, when achieved, play a central role in enabling the coordination of operator actions to handle out-of-the-ordinary events.

In the case of manual control, passengers on board will be notified via audio announcements over speakers. When manual control is engaged, the ferry will stay in position until the operator uses the joystick to manoeuvre the ferry or pushes a button to resume normal operation. The operator can change the camera view between fore and aft on the ferry and land-based cameras with zoom function. Passengers can also provide information about the situation on the ferry and its surroundings by using a two-way communication link through an HMI display onboard milliAmpere2.

#### Participants

An essential part of a CRIOP exercise is using experiences from similar control centres in operation and including the end-users as participants in the analysis. However, there is no SCC in operation for MASS yet, and we do not have an end-user (SCCO) in place at this point. It is still not agreed on what skills and qualifications are required for the SC operator. Findings in the HUMANE project (Lützhöft et al. 2019) point to the need for seafarer experience and the operator having certified navigational skills and seamanship. In our case, the operational domain of the autonomous ferry is limited to an urban canal, and it will not operate in harsh weather conditions. Still, the SCCO would need knowledge of the COLREGs rules and have a feeling for how a small ferry moves. Therefore, mariners with seagoing experience were invited to the workshop. In addition, we looked to other domains with remote control experience and invited participants from companies working with automated guided vehicles and autonomous shuttle buses. Furthermore, participants with system knowledge, including engineers and designers from the milliAmpere2 and Autoferry^2^ project teams, were also invited to the workshop.

The invited participants were selected through convenience sampling and the SCL network at NTNU. In total, 12 participants attended the workshop. The characteristics of the participants are listed in Table 3. The circles indicate the expertise area. Black circles indicate participants with at least five years’ experience; white circles indicate participants with less than five years’ experience. A letter is added to the participant number to indicate the gender of the participants: F is female, and M is male.

### The format of the case study 

The study was carried out during the COVID-19 pandemic, and the need for social distancing made the workshop subject to a digital solution. This had both positive and negative aspects, further discussed in Sect. 3.6. We used Microsoft Teams’ digital platform (Microsoft 2022) and the online whiteboard software Miro (miro 2022). Miro worked as a digital whiteboard for visual collaboration during the workshop (using digital “Post-it”-notes, adding comments and questions), collecting and analysing data after the workshop. The schedule and walkthrough process were presented with the STEP diagram and screenshots of the GUI showing the simulated scenario.

In the preparations for the workshop, a modification to the scenario analysis method was made. Due to a time constraint of two hours, the participants’ limited knowledge of CRIOP studies, and the fact that this was a first design iteration of the HMI, we chose not to follow a strict stepwise approach in the scenario analysis. The sequential events in the STEP diagram plus the following list of considerations were merged into one brainstorming process focusing on the following:

- Ask “what can go wrong?” (Identify hazards) and “what would the operator wish to do in each situation?” Use questions related to performance influencing factors and Hollnagel’s Simple Model of Cognition, such as “How is the SCCO notified? What information is presented? What happens if the information is not presented? How can the information be misunderstood? Which erroneous decisions can be made?”
- Identify weak points in the designed HMI.
- Identify mitigation actions by discussing existing barriers and missing barriers.

## Data collection

As presented in Fig. 4, the results were evaluated by a short debrief at the end of the workshop and by sending out a survey to each participant. The questions assess the analysis’s validity, reliability, credibility, and usefulness. We define these terms accordingly in Table 4.

**Table 4:** Definition of terms and questions in the questionnaire

Term	Definition	Question(s) in the survey
External validity of the workshop settings	Whether the method “actually did what it aimed to do” (Salmon et al. 2020)	1. Was the scenario realistic? 2. Based on the provided information, did you manage to identify hazards and assess risks?
Credibility (or internal validity)	When the results of the study mirror the views of the participants in the study: whether the participants believe the results are valid (Mills and Gabrielle 2010)	3. Do you believe the scenario analysis results (identified weak points and mitigating barriers) are valid?
Reliability	If the study results can be reproduced under a similar methodology (Joppe, 2000)	4. Are the results of the analysis confirmed by other similar studies?
Usefulness	Whether the participants found the scenario analysis to meet its goal of improving the design of the HMI Usefulness is one of the many dimensions that influence and contributes to a product’s usability (Trudel, 2021)	5. How did you succeed in understanding and predicting the safety issues/weak points in the HMI? 6. Do you find the method helpful in including the human element in a risk assessment of the prototype design? 7. Did you learn anything from the workshop? Can you tell us more about what?

### Results from the case study

**Fig. 8:**
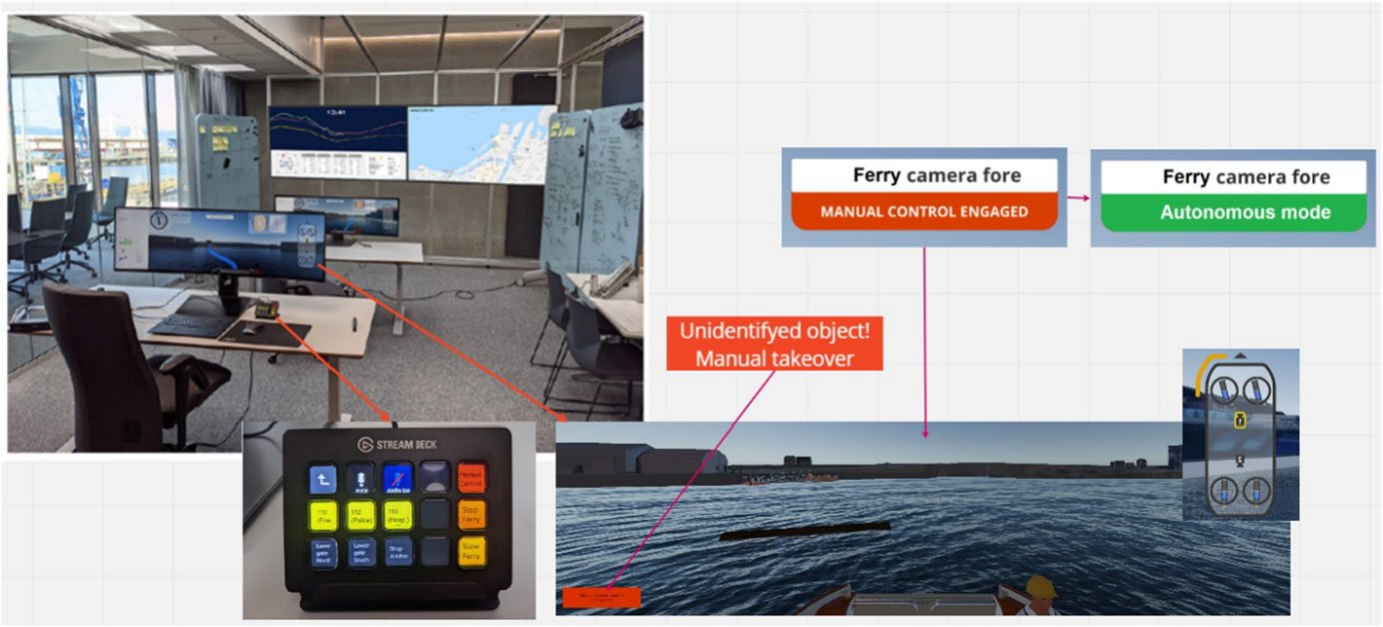
Screenshots of the simulated scenario in the GUI and the peripherals at the shore control centre

**Fig. 9:**
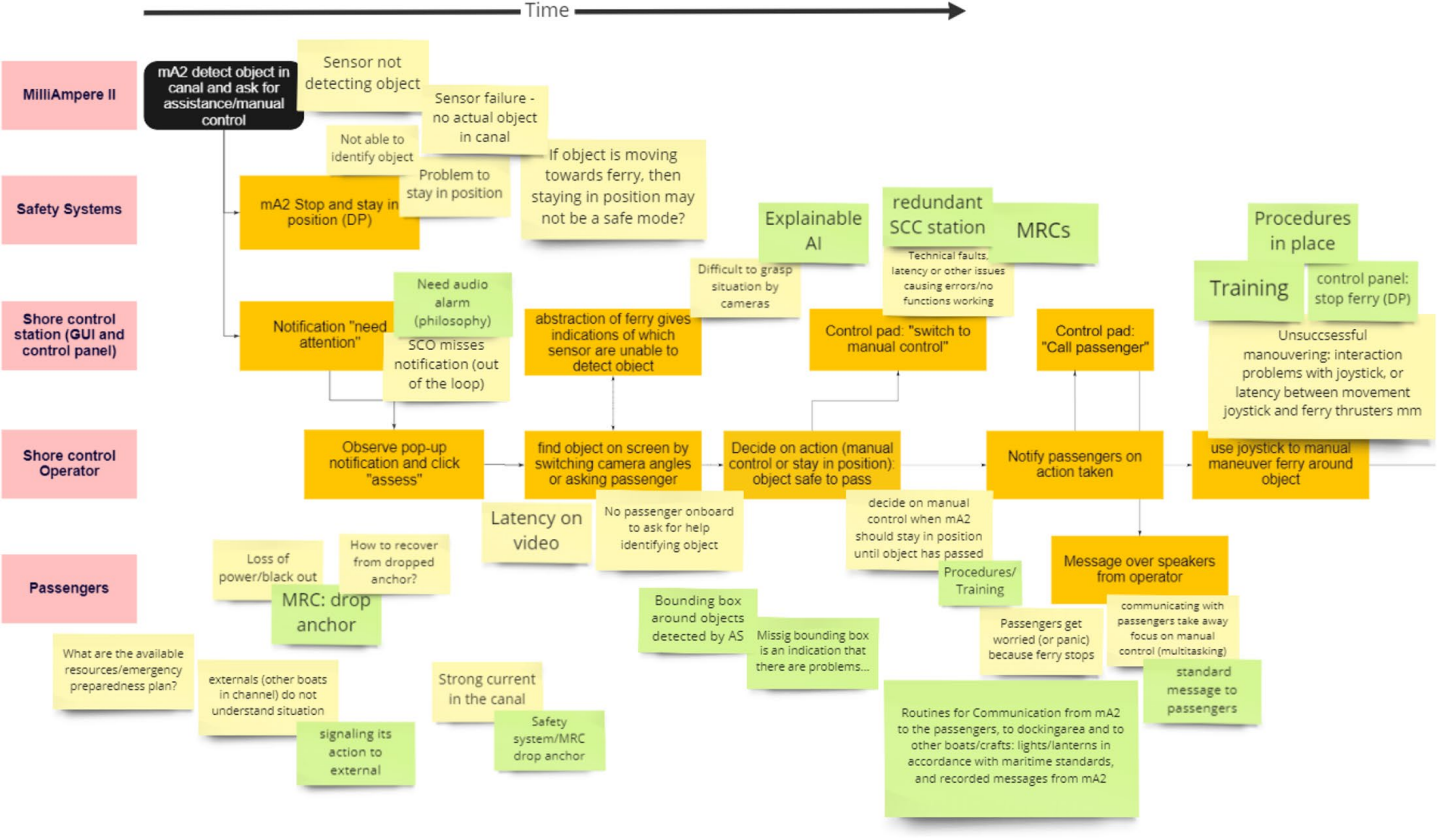
A extract of the STEP diagram from Miro after the workshop

The hazard identification part of the scenario analysis was convened in a brainstorming session after the participants became familiar with the concept and the scenario analysis process (including the STEP diagram). The facilitator wrote “Post-it”-notes based on input from the participants. The data collection of hazards, weak points, and mitigating measures added to the STEP diagram during the workshop can be found in Figs. 8 and 9 in Appendix 7.

For each event, the participants suggested hazards and how probable this was in the given context (the combination of which represented the event’s risk according to the classical definition) and discussed potential barriers to avoid or mitigate the hazards. The participants were encouraged to focus on the SCCO’s capabilities, tasks, sensemaking, and possible error sources and malfunctions in the HMI. They were allowed to drift around other topics, triggering discussions not directly related to the tasks and events in the STEP diagram, but instructed to not spend too much time on disagreements in assessing the severity of consequences or probabilities. Instead, they were encouraged to identify additional mitigating measures and discuss their potential effects.

From the discussion in the workshop and the identified risks and mitigating measures (barriers), the following identified weak points (design issues and safety problems) and mitigating actions (suggestions for improvements) can be summarised:

- The existing CONOPS does not address the responsibilities of the SCCO. The role of the SCCO is a missing priority! A list of situations where immediate SC intervention is required must be established:
- When and how should the operator intervene? Descriptions of tasks and supporting working procedures are needed.
- What are the needed skills and training for the SCCO?
- No alarm philosophy is established. Notifications on a screen alone are not enough.
- Recovering from a “safe state:”
- After going to a safe state and dropping the anchor, what happens?
- What are the available resources to pick up passengers and resume operation? An emergency preparedness strategy is missing.
- Related to high-performance HMI: “ easy to discover”-notification messages should appear centred on the main screen and not down in the left corner.
- Develop the GUI to support explainable artificial intelligence:
- Bounding box around detected object to avoid misunderstanding which object is within the collision zone and detected by the ferry.
  o Implement layers showing the collision zone when necessary.
- A safety management system must be established: how can the SCCO report incidents (an unplanned, uncontrolled event that under different circumstances could have resulted in an accident), near-accidents (an event that could reasonably have been an accident but did not, typically due to the SCCO intervening), and accidents (an unintended sequence of events that lead to harm to people, environment, or other assets)?
- More data from actual experiments are needed, i.e. systematic recording of accidents and incidents in the testing phase.
- Maintenance issues: How will the SCC handle this? How is the status of the technical systems presented to the SCCO?

There were also some “loose” “Post-it”-notes considering general hazards, questions related to the overall structure, responsibility gaps, and the CONOPS (see Fig. 9 in Appendix 7.). These essential issues may not have been revealed by analysing individual hazardous events and their consequences.

### The contextual conditions of the case study

The preconditions of the workshop gave some limitations to the applicability of the method:

- The autonomous passenger ferry milliAmpere2 was designed with a safety operator onboard. Hence, the CONOPS and preliminary hazard analysis were carried out with this precaution. In our case study, we provided a background document based on this CONOPS, but where the safety operator was transferred to the SCC and became a SCCO. The SCCO tasks and responsibilities were adjusted accordingly.
- An incremental approach was taken in this project. Ideally, the design of the SCC would be considered from the beginning of the project and not as an “add on” to be designed when the vessel and its technology are built and completed.
- Making retrofits to the ferry now will be expensive and challenging. Nevertheless, it is essential for good human system integration. Risks identified regarding communication, emergency preparedness, and other aspects may influence the final design, for example, the need for a two-way communication system and automatic fire detection and sprinkler system at the top side of the ferry. This is not in place on the milliAmpere2 today.

Ideally, we would have been physically present at the SCL experiencing the simulated scenario at the SC station. This would have made the scenario more tangible and closer to the actual operational environment. However, screenshots of the simulated scenario related to each event in the STEP diagram were presented to the participants. There were some benefits of having a digital workshop. Firstly, we were able to recruit participants located outside of the Trondheim area. We experienced that it was favourable to have a digital meeting, and we found it easier to get participants to spare two hours of their working hours when they could log on from their own office. During the workshop, the participants could (anonymously) type “Post-it”-notes and post them to events and tasks in the STEP diagram. Using the digital collaboration platform, Miro enabled us to more accessible collect data and document the process.

### Evaluation by the participants

At the end of the workshop, a round of “criticism of the method” revealed some practical implications of the organisation of the tabletop exercise, like involving experts in the selection of scenarios and better structuring of the brainstorming process. In the questionnaire sent out to the participants after the workshop, open-ended questions related to the method’s credibility, accuracy, reliability, and validity and results were asked (see Table 4). Based on the feedback from the participants and discussions among the authors, a summary of this, including potential future work to address identified threats and weaknesses, is listed in Table 5 in Appendix 8. The main feature mentioned by the participants was how the method provided a common platform for understanding the operations and how the SCCO could handle different situations. By visualising the scenario in a simulation of the HMI and structuring the discussion to events in a STEP diagram, the scenario became easy to comprehend. The method facilitated an open discussion and brainstorming around possible risks. Furthermore, the participants appreciated the possibility of exchanging experiences across disciplines and domains.

## Discussion

Risk is about more than expected values. Expected value decision-making can be misleading, especially in the design phases of MASS, where risk and safety might be best understood and communicated in ways other than probabilistic risk analysis. One such way is by understanding and assessing risk in terms of knowledge and lack of knowledge and by identifying hazardous events and their tangible effects. We have presented a method combining HCD and risk assessment elements. The scenario analysis fulfils several criteria for a suitable hazard analysis method as defined by Zhou et al. (2020). Valuable features of the method include its ability to highlight possible issues of the SCC concept, as well as uncertainties, knowledge gaps, and missing priorities. This provides valuable input to a revised and more detailed CONOPS and system architecture. The analysis can also reveal interdependencies between subsystems not revealed by other risk assessments, helping the team agree on how to solve an issue and contribute toward the overall aim of improving the safety of the MASS system. This is also supported by the results in the case study, where we identified a wide range of weak points when analysing how the HMI would work in practice. Most of the identified weak points were crucial questions to the developers and the organisation that need to be answered before a new design iteration to the next development phase. The existing risk analysis and preliminary hazard analysis did not identify these weak points (design issues and safety problems).

An example of a risk assessment focusing solely on the technical aspects of MASS is presented by Banda et al. (2019). Here, the authors apply the STPA but do not integrate a SCC. The SCCO is left out of the scope and is only mentioned when identified as a barrier against accidents as if an addendum to designing the autonomous ferries. This undervalues the potential of incorporating an understanding of human needs and capabilities early in the design process. STPA is a systems-theoretic approach used, among other things, to analyse human-automation interaction. Applied to the SCC case, this includes identifying any unsafe control actions performed by the human operator. However, where the analyst chooses to set the system boundaries will strongly affect the outcome of the analysis, as the mentioned study implies. The adapted scenario analysis in this paper explicitly addresses the interactions between SCCO and MASS. Hence, the most considerable improvement of the scenario analysis, compared to established practices, is the strong involvement of the SCCO. Involving the SCCO addresses the requirements concerning the “human element” in the IMO’s FSA and the interim guidelines for MASS trials.

A limitation is incurred by the want of SCCO taking part in the case study. At present, there are no certified SCCO, nor are no formal training standards available like there are for conventional seafarers. The CRIOP framework nonetheless explicitly states that the end-user must be included as a participant (Aas et al. 2009). This condition could only be partly met by selecting participants with relevant backgrounds, as judged by the workshop organisers. Inviting mariners with experience with highly automated bridges might be an option. However, this depends on the required qualifications (skills and education) and the responsibilities and tasks envisioned for the SCCO.

Our focus was on cognitive and not physical ergonomics related to workplace comfort and safety, typically included in an entire CRIOP exercise. The prototype was the first version of the initial design; hence, the complexity and fidelity of the analysis were consistent with the data and information we had available. The model (STEP-diagram) and analysis were of a high level and can be expected to mature in the following design phase. The systematic activities in a scenario analysis should be adjusted to the design phase in question.

Typically, a CRIOP study runs over several days, encompassing several critical scenarios (Johnsen et al. 2011). By contrast, our case study was more focused, and only one scenario was analysed. The method’s validity is already proven. It is considered a “best practice” tool in the design process and for validating and verifying control centres in the oil and gas industry. The validity of testing the applicability in our case study was evaluated in terms of participants’ feedback (summarised in Appendix 8.) and the method’s ability to identify hazards, risks, and issues. All participants accepted the scenario as possible, and a long list of hazards and weak points were identified. The method’s credibility is considered sufficient as the participants were recruited from different fields of expertise. None of the participants, except the facilitator, attended the preliminary hazard analysis. After the workshop, all participants reviewed the analysis report and confirmed that they believed the results were valid. In addition, several of the identified hazards and safety issues were mentioned in the preliminary hazard analysis carried out by zeabus. However, additional hazards were also identified. Threats to the validity, credibility, and reliability of the method are listed in Appendix 8. These are biases from the participants already involved in the HMI-design process, lack of having the actual end-user present, time constraints, and limited opportunities to modify the ferry’s design, configurations, and technical solutions. In the case study, we applied the method on a prototype of the HMI during the early preliminary design phase of a SCC interface. This led us to apply a semi-structured approach where we combined some of the activities in the scenario analysis. The main focus was on the group discussion of safety issues, hazards, and possible mitigating measures.

In the case study, the scope is limited to one operator. In a future SCC, there could be several SCC stations with one operator at each station monitoring a fleet of unmanned ferries. An adapted scenario analysis should, in this context, also consider fleet operations and team collaboration. In our case study, the scenario analysis did not include events that involved such team cooperation. One of the reasons for this was that the CONOPS did not specify SCC organisational design.

One of the advantages of the method lies in its ability to generate discussions between stakeholders with different backgrounds on human factors issues, risks, and possible mitigating measures. The participants do not need extensive expert knowledge to facilitate the analysis, nor do they need to go through many complicated steps. The simplicity of the STEP diagram also makes it quicker for participants to familiarise themselves with the scenarios. We may risk simplifying the scenario analysis when trying to model complex systems. The analysis aims to be easy to understand and produces results, but its reliability and quality might be questionable for complex problems. In many ways, human-centred risk-informed decision-making must find the balance between making the risk analysis practicable and providing a sufficiently comprehensive scenario analysis for demonstrating safety.

All risk assessments have limitations and should not be used mechanically. As Brown and Elms (2015) stress, our perception of risk is constructed and affected by a range of typical biases and fallacies. For the scenario analysis, as for most risk assessments, the results are highly dependent on the expertise and experience of the participants. Another bias is the limitation of using input from a brainstorming session that gives the participants time to think and reflect on what they should do in a scenario. By doing so, what they say they will do may not be the same as what they would actually do. The facilitator should also be aware that individual operators present in the same context at the same time (i.e. in a situation or event) may ascribe different meanings to it. Hence, there is no objectively correct interpretation of what may go wrong. Different interpretations and perceptions of risks should be appreciated (see Goodman and Kuniavsky (2012)).

The scenario analysis has not relied on quantitative measures but instead reached its conclusions based on the qualitative information and contributions from the participants. In the analysis, we walked through critical events related to the SCCO and identified hazards and safety issues, aiming to improve risk understanding, which will provide valuable decision support. By focusing on the capabilities of a SCCO, we are relating risk to performance. This aligns with recently recommended practices where risk thinking is combined with principles and methods of robustness and resilience (Aven 2016).

## Conclusions

Risk assessments can improve the understanding of the system, safety controls, and hazards of the activities under investigation. The traditional risk analysis methods applied in the maritime industry today may not be sufficient to address the complexity and emergent risk of MASS. Different risk analysis methods should be applied for different purposes at different phases of the design process. A risk analysis method focusing on human aspects should be used for risk-based design of MASS (DNV 2018). Examples of such methods are the CRIOP study, which can provide flexibility, and explainability and highlight safety issues by detailed identification of weak points when applied to an HMI design process. We have presented a qualitative case study of an interdisciplinary human-centred risk assessment method. The method is inspired by the scenario analysis in the CRIOP Framework. In this paper, we asked whether an adapted version of the scenario analysis could offer a helpful tool for supporting human-centred risk-informed decision-making in the design of a SCC.

The case study shows that the method could be applied for risk-informed decision-making in the design phase of SCC for MASS operation. In the case study workshop, a scenario of a handover situation where the simulated autonomous system asks for assistance from the SCCO was presented. The case study was carried out on a digital platform. Twelve people, including the design and engineering team of four, attended the workshop.

Findings from the study show that the scenario analysis method can be a valuable tool to address the human element in risk assessment by focusing on the operators’ ability to handle the situation. Unlike traditional hazard analysis tools, the method is especially useful in identifying HAI-associated hazards. The method is cross-disciplinary and can be an arena for learning and sharing experiences. The simplicity of the method encourages an open discussion and involvement of several actors. Good design practice utilises human factor knowledge that emerges from the users sharing their experiences. The results reveal that the scenario analysis method could minimise the gap between WAI and WAD.

The experience of using the scenario analysis method for the evaluation and validation of control centres in the Norwegian oil and gas industry has been positive (Aas et al. 2009). In our study, the scenario analysis gave the workshop a necessary and efficient structure to analyse and discuss risks and mitigating measures. Hence, the analysis supports risk-based design for the human control element in autonomous ferries, allowing for human in the loop-capabilities. The design of an HMI supporting a safe and dynamic transition between autonomous and manual mode is a critical prerequisite for their implementation in urban waterways.

### Further work

Based on the feedback on the issues of credibility, validity, and reliability of the scenario analysis (listed in Appendix 8.), there is a need for improved method guidance. Guidelines for the application at different phases of the design process should be developed, which is also a recommendation in a report on “Human-centred design and HMI in the development and implementation of autonomous systems in drilling and well” by (Johnsen et al. 2020). The performance shaping factors, checklists, and guidewords for the scenario analysis should be updated and specified for MASS operation.

With the increased opportunities and benefits of using simulations, a scenario analysis could benefit by having the scenarios presented in a simulation. The simulation would provide the participants with a more realistic understanding of the situation, different actions could be tested, and the scenario could be “paused” for elaboration on specific issues. The method should be applied to several cases and scenarios to increase its validity.

## Appendix 1

Figure 9 below is a modified screenshot of a part of the STEP diagram in the digital platform Miro. In the STEP diagram, the involved actors (denominates a person or object that affects the event (Johnsen et al. 2011)) are listed vertically, while the events are textboxes placed according to the order in which they occur. Arrows illustrate their relationship (causal links).

“Post-it”-notes were added during the workshop. Yellow “Post-it”-notes indicate hazards and safety issues, while green “Post-it”-notes indicate mitigating measures and possible solutions. The “Post-it”-notes are adjusted for better readability.

## Appendix 2

**Table 5:** Summary of the scenario analysis methods’ purpose and issues of credibility, validity, and reliability, including potential future work to address identified threats/weaknesses

	General for the scenario analysis	Specific for the case study
Purpose and intent	The purpose depends on the context and design phase. The overall goal is to verify that the operator can perform the task at hand, considering cognitive abilities, human-system interactions, and other performance shaping factors	Risk analysis of the preliminary designed HMI to get input on a safe design of an SCC
External validity	The scenario analysis allows for tractability by employing a modelled scenario, making the actors, subsystems, and interactions visible to the analysts The open questions allow for a greater level of discovery	All participants accepted the scenario as possible. Background information was provided. Participants unfamiliar with SCC and the operator’s tasks were given time to ask clarifying questions. A long list of hazards and weak points were identified
Threats to validity	In a complex (intractable) system like MASS, we would never identify all scenarios of interest. There are limitations to the Scenario Analysis as a risk assessment Not having the necessary knowledge of the systems to be assessed	There was a lack of detailed information on some technical solutions in the early design phase. Hence there is a risk for omissions, i.e., failing to identify crucial hazards and/or weak points
Future work to address validity	The validity (including limitations of the assessment) must be addressed and explained to the decision-makers as a part of the risk communication	
Credibility	The scenario analysis depends on the context. The scenarios to be analysed can be retrieved from a preliminary hazard identification. Identifying the risks by using different techniques increases the validity of the results	Participants were recruited from different fields of expertise. Engineers, interaction designers, human factors experts, and participants with seafarer experiences were part of the analysis group The participants believed the results were valid. Participant check: The analysis report was taken back to the participants to be confirmed and to evaluate the method. In this way, the plausibility and truthfulness of the analysis were recognised and supported
Threats to credibility	Not having the “right” participants. The strength of the results depends on the expertise/knowledge of the participants	Biases: Some participants had already made decisions when designing the conceptual HMI The time constraint limited the brainstorming process causing potential valuable input not to be considered
Future work to address credibility	Select the participants carefully Give the participants sufficient time to write “Post-it” notes and post these anonymously Furthermore, develop the digital platform for the analysis. The facilitator should create a good atmosphere and aim to be non-judgmental and not interrupt	
Reliability	The method is well-known in the oil and gas industry (Johnsen et al. 2011). It is seen as a “best practice” solution for integrating Human Factors into the design process The purpose is not to attain the same results but to provide a methodological approach to identify hazards and weak points	The 12 participants came from various backgrounds: a cross-disciplinary group of hardware designers, human factors experts, programmers, engineers, and people with experience as seafarers Several of the identified hazards and safety issues had been briefly mentioned in the Preliminary Hazard analysis carried out by zeabus
Threats to reliability	The results of an analysis are highly likely to vary depending on the participants and the design phase If the STEP diagram is confusing and does not provide the participants with the proper explanation of the events, the quality of the analysis will be poor	The analyst team did not include the actual end-user, the SCCO The time constraint and limited knowledge of the system architecture were mentioned as a challenge for the participants
Further work to address reliability	Make sure to invite the “right” participants. Have sufficient and updated CONOPS Involvement of experts in selecting critical scenarios and making the STEP diagram	
Usefulness	The method can fill an “including the end-user” gap by providing a human (end-user) centred approach to the risk assessment In studies applying the method in the oil and gas sector (Aas et al. 2009), users reported an increased understanding of the perspectives, needs, and requirements of the control room operators and potential hazards in the socio-technical system	Many participants reported that they could easily understand the scenario making it easy to discuss what-if questions, address hazards and evaluate the preliminary HMI design Discussions between the programmers and the hardware designers on how the operator would handle surprises helped meet the goal of getting input on a safe(r) designed solution. Several participants reported that they had learned a new framework and considered it a supportive tool for risk assessment in the design phase
Threats to usefulness	Cost/benefit of the time/resources spent on the analysis The usefulness of qualitative risk assessments to support defining a specific safety level is challenging Whether the analysis generates enough information for the decision-makers depends on selected scenarios and the quality of the risk assessment	One participant questioned why the passenger ferry was in the final construction phase while the SCC was in the design phase. This limits the opportunity to modify the design and technical solutions Several participants stressed the need for Scenario Analysis of several scenarios
Further work to address the usefulness	Need for better method guidance: The scenario analysis’s performance shaping factors, checklists, and guidewords should be updated and specified for MASS operation (primarily focusing on navigational aspects)	

## Footnotes

1 MilliAmpere2 is a full-scale prototype of the world’s first autonomous passenger ferry, milliAmpere, designed to become a living lab in Trondheim city, with capabilities and supporting infrastructure enabling trial passenger operation. https://zeabuz.com/miliampere/

2 A cross-disciplinary research project at NTNU on autonomous all-electric passenger ferries for urban water transport https://www.ntnu.edu/autoferry
